# Characteristics of Interstitial Fibrosis and Inflammatory Cell Infiltration in Right Ventricles of Systemic Sclerosis-Associated Pulmonary Arterial Hypertension

**DOI:** 10.1155/2010/604615

**Published:** 2010-09-30

**Authors:** Maria J. Overbeek, Koen T. B. Mouchaers, Hans M. Niessen, Awal M. Hadi, Koba Kupreishvili, Anco Boonstra, Alexandre E. Voskuyl, Jeroen A. M. Belien, Egbert F. Smit, Ben C. Dijkmans, Anton Vonk-Noordegraaf, Katrien Grünberg

**Affiliations:** ^1^Department of Pulmonary Diseases, VU University Medical Center, VU University Amsterdam, De Boelelaan 1117, NL 1007 MB Amsterdam, The Netherlands; ^2^Department of Pathology, VU University Medical Center, VU University Amsterdam, De Boelelaan 1117, NL 1007 MB Amsterdam, The Netherlands; ^3^Department of Cardiac Surgery, VU University Medical Center, VU University Amsterdam, De Boelelaan 1117, NL 1007 MB Amsterdam, The Netherlands; ^4^Department of Rheumatology, VU University Medical Center, VU University Amsterdam, De Boelelaan 1117, NL 1007 MB Amsterdam, The Netherlands

## Abstract

*Objective*. Systemic sclerosis-associated pulmonary arterial hypertension (SScPAH) has a disturbed function of the right ventricle (RV) when compared to idiopathic PAH (IPAH). Systemic sclerosis may also affect the heart. We hypothesize that RV differences may occur at the level of interstitial inflammation and—fibrosis and compared inflammatory cell infiltrate and fibrosis between the RV of SScPAH, IPAH, and healthy controls. *Methods*. Paraffin-embedded tissue samples of RV and left ventricle (LV) from SScPAH (*n* = 5) and IPAH (*n* = 9) patients and controls (*n* = 4) were picrosirius red stained for detection of interstitial fibrosis, which was quantified semiautomatically. Neutrophilic granulocytes (MPO), macrophages (CD68), and lymphocytes (CD45) were immunohistochemically stained and only interstitial leukocytes were counted. Presence of epi- or endocardial inflammation, and of perivascular or intimal fibrosis of coronary arteries was assessed semiquantitatively (0–3: absent to extensive). *Results*. RV's of SScPAH showed significantly more inflammatory cells than of IPAH (cells/mm^2^, mean ± sd MPO 11 ± 3 versus 6 ± 1; CD68 11 ± 3 versus 6 ± 1; CD45 11 ± 1 versus 5 ± 1 , *P* < .05) and than of controls. RV interstitial fibrosis was similar in SScPAH and IPAH (4 ± 1 versus 5 ± 1%, *P* = .9), and did not differ from controls (5 ± 1%, *P* = .8). In 4 SScPAH and 5 IPAH RV's foci of replacement fibrosis were found. No differences were found on epi- or endocardial inflammation or on perivascular or intimal fibrosis of coronary arteries. *Conclusion*. SScPAH RVs display denser inflammatory infiltrates than IPAH, while they do not differ with respect to interstitial fibrosis. Whether increased inflammatory status is a contributor to altered RV function in SScPAH warrants further research.

## 1. Introduction

Systemic sclerosis (SSc) is a disease with a multifaceted pathology which is characterized by an enhanced inflammatory status, vasculopathy, and excessive fibrosis in skin and internal organs [1]. Pulmonary involvement, either lung fibrosis or pulmonary arterial hypertension (PAH), is the leading cause of death in SSc [1]. SSc complicated by PAH (SScPAH) carries a mortality of 50% at 3 years, which is higher when compared to idiopathic PAH (IPAH) [2–4]. 

These differences might be explained by comorbidity due to the systemic nature of SSc, a higher age of onset of disease, and differences in pulmonary vasculopathy [5, 6]. There are also indications that the RV in SScPAH adapts differently as compared with IPAH patients: several studies have shown lower pulmonary arterial pressures in SScPAH than in IPAH, while cardiac index and pulmonary vascular resistance were similar [6–10]. Additionally, it has been demonstrated that RV pump function is different in SScPAH when compared to IPAH [10]. Furthermore, a different response to cardiac load has been suggested by the disproportionate levels of N-terminal probrain natriuretic peptide found in SScPAH compared with IPAH, despite the less severe hemodynamic abnormalities [7, 11].

There is no knowledge concerning the etiology of altered RV function and adaptation in SScPAH. Here, for the first time, histolopathologic characteristics of the RV in a well-defined SScPAH group will be explored, focusing on inflammation and fibrosis, two main pathologic features of SSc. Comparison is made with RVs from IPAH patients and healthy controls. It is hypothesized that inflammatory status and fibrosis are quantitatively different in the RV interstitial myocardium of SScPAH patients as compared with that of IPAH patients. To test this hypothesis, we determined the numbers of macrophages, lymphocytes, and neutrophilic granulocytes and quantified fibrosis in the myocardial interstitium of RV's of SScPAH and IPAH patients. To evaluate an effect of pressure overload from the lesser circulation in PAH, RV interstitial fibrosis and interstitial inflammation were compared to the left ventricle (LV). Hearts from healthy individuals that died acutely of traumatic causes served as controls.

## 2. Methods

### 2.1. Patient Characteristics

The cases examined in this study were retrieved from the departments of pulmonary diseases of the VU University Medical Center, Amsterdam, the Netherlands. The study was approved by the Institutional Review Board on Research Involving Human Subjects of the VU University Medical Center.

Patients who had been treated for PAH between 1998 and 2007, of whom cardiac tissue obtained at autopsy was available, were deemed eligible for the study. General patient characteristics are described in Table 1. The diagnosis of SSc (as established by a reumathologist), SScPAH, and IPAH was verified by reviewing the medical records, including lung function data at baseline as well as HRCT studies. Patients with restrictive disease, as indicated by total lung capacity as percentage of predicted (TLC%) <70%, vital capacity (VC%) <70% and/or severe fibrosis on HRCT scan, were classified as pulmonary hypertension due to restrictive disease and therefore excluded from this study. SSc classification, SSc disease duration, and antibody profile were recorded (Table 2) [12, 13]. Of the SScPAH group, 3 patients had died of RV failure, one had died of hypovolumic shock due to iatrogenic intra-abdominal bleeding after ascites drainage which was caused by right heart failure, and one died postoperatively within 2 days after lung transplantation. Eight IPAH patients had died of RV failure and one of hemorrhagia from the arteria pulmonalis.

The hearts from four patients who had acutely died from traumatic, noncardiopulmonary, noncerebral causes and who did not have a cardiopulmonary medical history, served as healthy controls.

### 2.2. Tissue Preparation and Immunohistochemistry

#### 2.2.1. Inflammation

Serial adjacent sections of myocardial tissue (4 *μ*m thick) were deparaffinised for 10 minutes in xylene at room temperature and dehydrated through ascending concentrations of ethanol. Endogenous peroxidase activity was blocked by incubation in 0.3% (v/v) H_2_O_2_ in methanol for 30 minutes. Tissue sections were subjected to antigen retrieval by boiling in 10 mM sodium citrate buffer, pH 6.0 for 10 minutes in a microwave oven. All antibodies and normal serum were diluted in PBS containing 1% (w/v) bovine serum albumin (BSA). Tissue sections were preincubated for 10 minutes with normal rabbit and normal swine serum (1 : 50), followed by incubation for 1 hr with either polyclonal rabbit antihuman myeloperoxidase (MPO) (Dako, A0398, Denmark, 1 : 50 dilution), or mouse monoclonal antibody CD68 (KP1) (Dako, M0814, Denmark, 1 : 400 dilution) and mouse monoclonal anti-human CD45 (Dako, M0701, Denmark, 1 : 50 dilution) antibodies. After washing in PBS, tissue slides were incubated for 30 minutes with a biotin-conjugated secondary antibody rabbit anti-mouse biotin (1 : 500 dilutions) and swine anti-rabbit biotin (1 : 300 dilution) (Dako, A0063, Denmark), followed again by washing in PBS. Then, slides were incubated with streptavidin-biotin complex (sABC; 1 : 1000 dilutions) for 1 hour. Finally, primary antibodies were visualized with 3,3′-diaminobenzidine (DAB; 0.1 mg/ml, 0.02% H_2_O_2_). Slides were counterstained with hematoxylin and mounted with Depex and a coverslip.

As a negative control, the primary antibody was replaced by phosphate-buffered saline or an irrelevant antibody; these heart tissue slides were found to be negative.

#### 2.2.2. Fibrosis

In order to determine the amount of interstitial fibrosis, paraffin-embedded sections of myocardial tissue (4 *μ*m thick) were stained with Picrosiriu-red. Sections were incubated for 10 minutes in xylene at room temperature to remove paraffin and transferred to water through descending concentrations of ethanol (100%, 96%, 80%, and 70%, all 10 s). Staining was performed using 0.1% solution of Sirius red F3BA in saturated aqueous solution of picric acid for one hour at 25°C [14, 15]. Subsequently, sections were differentiated in 0.01N HCl for 2 minutes. Sections were dehydrated in ascending concentrations of ethanol (70%, 80%, 96%, and 100%, each 10 seconds) and cleared in two stages in xylene, 10 minutes each. Sections were covered with Entellan mounting medium (Merck, Darmstadt, Germany) and a glass cover slip. In addition, to determine the presence of intimal fibrosis in coronary arteries and arterioles, adjacent sections were stained for Elastica von Gieson (EvG).

### 2.3. Morphometric Analyses

#### 2.3.1. Inflammatory Cells

In each tissue slide of the RV and LV, intramyocardial areas were randomly chosen, with a minimum area of 15 mm^2^. In these areas, the number of extravascular neutrophilic granulocytes (MPO^+^), macrophages/histiocytes (CD68^+^) and lymphocytes (CD45^+^) was counted (Figure 2). Intravascular inflammatory cells were excluded. The average number of intramyocardial, extravascular inflammatory cells per mm^2^ was then calculated as the total score for each specimen according to Begieneman et al. [16]. All cell counts and scorings were done by two investigators (MJO, KTBM) who were blinded to the clinical diagnosis. In order to minimize interobserver variability, both investigators counted a training set of 9 different histological samples (3 of each staining). The inter-observer variation was <10% with a correlation of *r* ^2^ = 0.98. The presence of perivascular infiltrates and inflammatory infiltration of the endocardium and the epicardium was scored on a 4 point scale, ranging from 0 (absent) to 3 (extensive).

#### 2.3.2. Fibrosis

**Figure 1:**
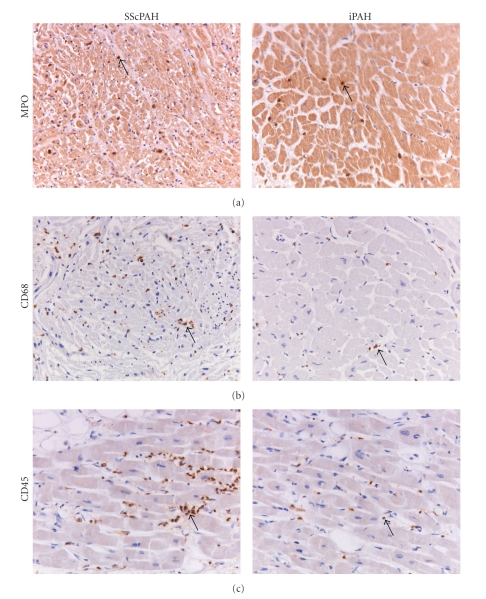
Sections of myocardial tissue stained with antibodies against (a) neutrophilic granulocytes (MPO positive), (b) macrophages (CD68 positive), or (c) lymphocytes (CD45 positive). Arrows indicate positive cells.

Picrosirius-red stained sections of paraffin-embedded cardiac biopsies were scanned in total with Mirax scan system (Zeiss, AG Germany). Areas were randomly selected from the digitised slides at a 20x magnification, covering at least 15% of the total area (Figure 1). Care was taken not to include vessels into the selected areas to ensure only interstitial myocardial fibrosis was quantified. Interstitial myocardial fibrosis was assessed in the RV and LV, using a fully automated analysis according to Mouchaers [17]. 

In addition, the entire sections were, semiquantitatively, investigated for epicardial and endocardial fibrosis and scored on a 4 point scale ranging from 0 (absent) to 3 (extensive). Likewise, EvG-stained sections were used to score the presence of replacement fibrosis, as defined by fibrotic areas within the myocardium coincident with a loss of cardiomyocytes as well as for perivascular and intima fibrosis of coronary arteries/arterioles. 

### 2.4. Statistical Analysis

For the quantification of interstitial fibrosis and interstitial inflammatory cells, 5 samples of myocardial tissue from the RV and 4 from the LV of SScPAH patients were analyzed; 9 samples were available of both the RV and LV from IPAH patients and 4 samples from controls. Mann-Whitney *U* was used to determine differences between groups. All histochemical data are presented in graphs as median (range) and patient characteristics in tables as mean ± SEM. Fisher's exact test was used for comparison of infiltration of inflammatory cells of the endo- and epicardium between groups. *P* < .05 was considered statistically significant. 

## 3. Results

### 3.1. Patient Characteristics and Haemodynamics

Patient characteristics are listed in Tables 1 and 2. SScPAH and IPAH groups did not differ with respect to mean age. Mean survival of the SScPAH patients was significantly shorter compared to IPAH patients. Haemodynamic parameters at diagnosis were not different between the groups. However, SScPAH patients tended to have a lower mean pulmonary artery pressure as compared with the IPAH patients. DLCO in the SScPAH group was significantly lower as compared with the IPAH group. Two patients in the SScPAH group and 4 in the IPAH group had been treated with aldosteron antagonists or ACE-inhibitors. None of the patients had systemic hypertension.

### 3.2. Inflammation

**Figure 2:**
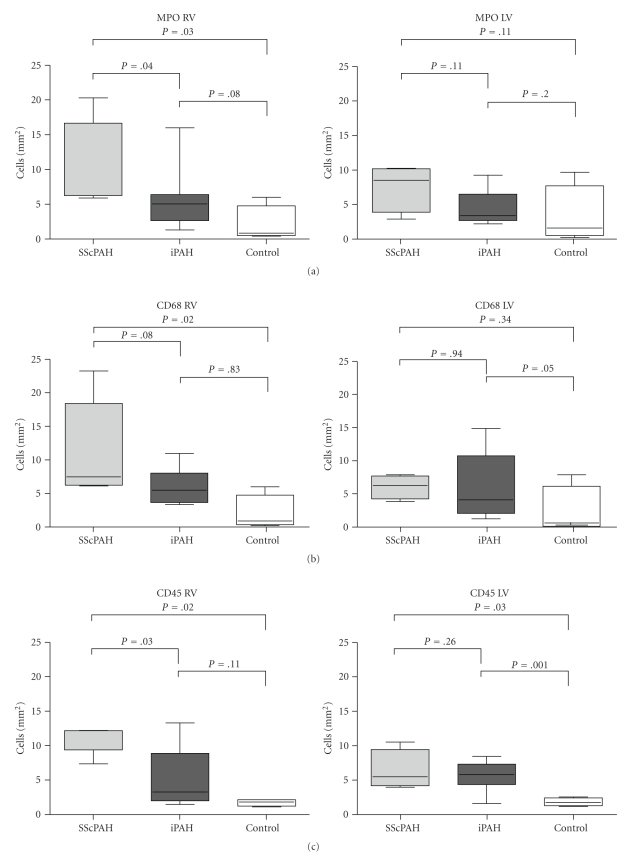
The number of (a) myeloperoxidase positive cells, (b) CD68 positive cells, and (c) CD45 positive cells was determined in the RV and the LV of systemic sclerosis-associated pulmonary arterial hypertension (SScPAH), idiopathic pulmonary arterial hypertension (IPAH) patients and in control subjects. Median and range are shown.

The RV's of SScPAH showed significantly more interstitial MPO- and CD45-positive cells when compared to IPAH. The numbers of MPO-, CD68- and CD45-positive cells/area were also increased when the SScPAH RV's were compared to normal controls (Figures 2(a), 2(b), and 2(c), and examples of immunohistochemical stainings are shown in Figure 1). In the RV of IPAH versus normal controls, no significant differences observed. In the LVs of SScPAH and IPAH, there were no significant differences in the number of inflammatory cells either. In SScPAH LV's, significantly more CD45 positive cells were observed as compared to normal controls, but no such differences were found for MPO nor for CD68. IPAH LV's demonstrated significantly more CD68 and CD45 as compared with normal controls. 

Infiltration of the endocardium and epicardium was not different between the SScPAH and IPAH, nor between RV or LV, for neither cell type (not shown). In all ventricles, a mild perivascular infiltration was observed, but no transmural infiltration of the vessel wall suggestive of vasculitis.

### 3.3. Fibrosis

**Figure 3:**
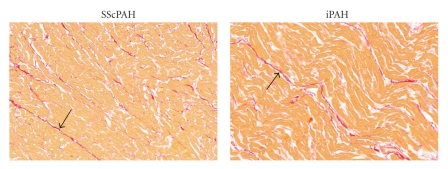
Representative samples of picrosirius red-stained myocardial sections of the RV of SScPAH and IPAH patients, used for quantification of interstitial fibrosis. Arrows indicate the red-coloured strains of fibrosis.

**Figure 4:**
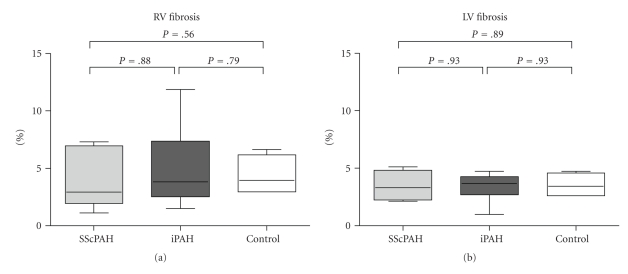
Quantification of picrosirius red staining in the RV of SScPAH, IPAH, and control subjects in (a) the RV and (b) the LV. Median and range are shown.

Representative samples of picrosirius red-stained sections, used for quantification of interstitial fibrosis, are depicted in Figure 3. Interstitial fibrosis in the RV was not different between the SScPAH and IPAH groups (Figure 4). LV interstitial fibrosis did not differ between the three different groups either. Focal epi- and endocardial fibrosis was seen in all subjects. 

**Figure 5:**
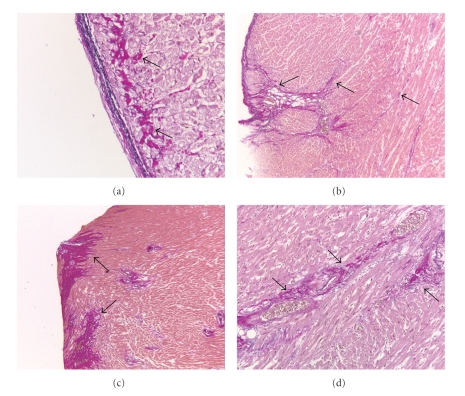
Representative samples of Elastica von Gieson stained myocardial sections of the RV of SScPAH patients, used for studying fibrosis in detail. (a) Some RV's of SScPAH patients revealed a pattern patchy replacement fibrosis, mostly localized subendocardially. This was also seen in some hearts of IPAH patients, but not observed in control. (b) In few cases, a pattern of strands of collagenous fibrous tissue surrounding microvasculature was observed, radiating from the epicardial coronary arteries to the subendocardial myocardium. (c) In some cases small infarcts in both SSc and IPAH hearts were observed. (d) An increase in perivascular fibrosis in some SScPAH hearts and in 1 IPAH heart was observed. All these observations were not made in control hearts.

On EvG-stained sections we analysed putative foci of replacement fibrosis. This was observed in 4 out of 5 RV's from SScPAH patients and in 5 out of 8 RV's from IPAH patients. The LV demonstrated replacement fibrosis in 2 out of 4 SScPAH patients and 5 out of 8 IPAH patients. In most cases, this fibrosis was patchy, showing microscopic foci, mostly localised subendocardially (Figure 5(a)). In few cases, a pattern of perivascular fibrosis of the microvasculature was observed, radiating from the epicardial coronary arteries to the subendocardial myocardium, ending in microscopic fibrotic foci (Figure 5(b)). In some cases, small infarcts (observed at gross pathology) were observed (1 SScPAH RV, 2 SScPAH LVs, and 2 IPAH RVs) (Figure 5(c)). This was not observed in hearts of control subjects. Finally, we investigated the occurrence of intimal fibrosis in intramyocardial coronary arteries and arterioles. This was observed in SScPAH in 1 RV and 3 LVs and IPAH in 1 RV. Perivascular fibrosis and adventitial remodeling was equally observed in both SScPAH and IPAH right and left ventricles (Figure 5(d)). This was not different from controls.

## 4. Discussion

In this paper, for the first time, histopathologic features of fibrosis and inflammatory status are described in the interstitial myocardium of the RV in a well-documented SScPAH group. As RV's of SScPAH patients have worse function than IPAH RV's, comparison took place with IPAH RV's. We observed significantly more extravascular inflammatory cells in the myocardial interstitium of the RV of SScPAH as compared with the RV of IPAH and as compared with normal controls. No significant difference in this respect was found between IPAH RV's and control RV's, nor between the LV's of both PAH disease groups. Interstitial myocardial fibrosis in the RV did not significantly differ between SScPAH and IPAH, nor between the PAH disease groups and normal controls. No differences were found with respect to interstitial fibrosis for the LV either. Although PAH patients had more replacement, perivascular, and subendocardial fibrosis when compared to controls, no differences were found between SScPAH and IPAH patients.

The presence of inflammatory cells in (interstitial) myocardial tissue in SScPAH has not been described previously. Two SSc cases with clinical LV failure, but without signs of increased RV afterload, have been described, demonstrating an increase in T-cells and CD68 positive cells in endomyocardial biopsies of the RV. [18] In IPAH, interstitial inflammatory cell infiltration in the RV did not differ significantly from normal controls in a previous report [16], which is in agreement with the present findings.

Fibrosis in hearts of SSc patients has been shown in autopsy studies, and tended to be patchy and distributed throughout all levels of the myocardium of the RV and LV [19–23]. In endomyocardial biopsies of RV's of SSc patients, Fernandes et al. [24] found increased collagen deposition as compared with normal controls. None of the above described studies included patients with confirmed pulmonary (arterial) hypertension. A recent study on cardiac MRI features in 52 SSc patients described delayed contrast enhancement, indicating the presence of myocardial fibrosis, in 1 of 8 SScPAH patients [25]. In IPAH, fibrosis in endomyocardial biopsies of the RV has been reported to be “mildly” increased, however, quantification nor specification concerning location was reported [26]. 

The study is limited by the small sample size. Despite this, the examined SScPAH RV histology is unique and has not been subject of study previously, set apart from the SSc group as a whole. Special care was taken to include only cases in which both the diagnosis of SSc and PAH was unequivocal, so as to optimize homogeneity, and thereby optimize statistical power. We therefore think that the exploratory data presented here provide relevant insight, warranting further study. An inherent limitation of this study is the use of archival autopsy material, which is shared by previous studies on this topic. As practically all patients died of PAH-related causes, the pathology in this series represents end-stage disease. Consequently, its features might differ from earlier and subclinical phases of the same disease. Also, sampling and processing of the RV and LV is often not done in a standardized way over the years. As little is known about uneven distribution of either fibrosis or inflammation, it is unknown as to whether this is a limitation to the study. For the immunohistochemical stainings, an internal positive control was present in all slides, and staining intensity as a possible consequence of differences in processing was irrelevant. The observation that even in end-stage disease significant differences were found in inflammatory cells suggests at least different pathogenic pathways. Our measurements included all intramyocardial areas (such as subepicardium, endocardium, or midwall area), and therefore the results depict the overall amount of interstitial fibrosis within the myocardium. Additionally, global scanning of the samples did not reveal differences between midwall and subepi-endocardial areas. This issue might be relevant as studies on cardiac MRI with delayed contrast enhancement suggest the mid-wall area as the predilection area in the majority of SSc patients who demonstrated DCE [25, 27, 28]. However, these studies did not describe DCE-uptake in the RV and results could not be histologically confirmed. One patient died within 2 days after lung transplantation. The short-term effects on RV of a sudden afterload normalization at histopathologic level are not known. It is known that the RV undergoes significant RV-remodelling within 3 to 6 months after lung transplantation as demonstrated by MRI [29]. We do not think that the RV in this patient morphologically underwent major changes. 

How do we interpret the elevated numbers of interstitial inflammatory cells in the RV of SScPAH hearts compared to IPAH? Mechanical stress due to RV pressure overload could be responsible for this finding as such a difference was not found when the LV's of both disease groups were compared. Indeed, increased mechanical stress may induce cytokine expression (e.g., monocyte chemoattractant protein-1 (MCP) and interleukin (IL)-8) in several cell types such as endothelial cells and cardiomyocytes [30, 31] through production of reactive oxygen species (ROS) with secondary activation of NF-*κ*B [32]. 

Increased susceptibility to ischemia, a known trigger to induce inflammatory cytokines, might also explain increased inflammatory cell infiltration in SScPAH, as structural and functional abnormalities of the small coronary arteries are known features of SSc [33–37]. However, in the present study we did not find differences in the presence of arteriolar intimal fibrosis between SScPAH and IPAH RV's. 

It is unclear whether the inflammatory cells are innocent bystanders or whether they play an active role in altered RV function in SScPAH. Most links between inflammation and a reduced contractility in chronic heart diseases have been forged on the basis of cytokines and chemokines [38–43] but there are limited data on the effect of cardiac tissue injury by neutrophils. In addition, it is known that macrophages can directly impair contractility of individual cardiomyocytes [44–48].

A significantly higher CD45-positive cell infiltration was found in the LV's of both SScPAH and IPAH as compared with controls, which is difficult to to explain. Increased CD45 infiltration of the myocardium is seen in viral myocarditis [49], however, in none of the patients other signs of myocarditis, such as necrosis or degradation of myocytes along with lymphocytic infiltrates, was seen.

We found no quantitative differences of interstitial fibrosis between SScPAH and IPAH. This, however, does not exclude the possibility that the composition or structure of the extracellular matrix components is different in SScPAH, which may result in different effects on ventricular function [47, 50, 51]. In agreement with previous reports, we did observe patchy and moderate replacement fibrosis in several hearts of SScPAH patients, both in the RV and LV [22, 52–54]. Replacement fibrosis is the end-result of either inflammation mediated and/or ischemia-mediated damage [55]. It is not clear in this study which mechanism predominates in SScPAH. As replacement fibrosis was also observed in IPAH patients, but not in control hearts, the focal fibrosis may ultimately be the result of increased RV pressure overload in pulmonary hypertension, regardless of its cause.

In conclusion, the present paper shows an increased number of inflammatory cells in the RV myocardial interstitium in SScPAH as compared with IPAH. No differences in (interstitial) fibrosis between the groups were found. Further research is warranted to evaluate the significance of these findings for the RV function in SScPAH patients.

## Figures and Tables

**Table 1:** General patient characteristics.

	SScPAH	IPAH	Control
	**N** = 5	*N* = 9	*N* = 4
Age, yrs	47 ± 4	47 ± 4	31 ± 4
Male/Female no.	1/4	2/7	—
Survival	1.1 ± 0.5	3.7 ± 0.9	—
Heart rate	85 ± 3	78 ± 5	
mPpa, mmHg	46 ± 7	62 ± 4	
PCWP, mmHg	6 ± 2	5 ± 2	
PVR, dynes·s·cm^−5^	1221 ± 691	1157 ± 144	
CI, l/min*·*m^2^	2.3 ± 0.9	2.3 ± 0.5	
Systolic blood pressure	105 ± 2	118 ± 8	
Diastolic blood pressure	69 ± 6	73 ± 5	
TLC, %	89 ± 5	92 ± 5	
DLCO, %	42 ± 6	64 ± 6	
Therapy at time of death			
Prostacycline (*n*)	4	7	
ERA (*n*)	1	2	
PDE-5 inhibitor (*n*)	1	0	
ABS		1	

Values expressed as mean ± SE or otherwise as stated. Abbreviations: ABS: atrial balloon septostomy;   CI: cardiac index; DLCO%: percentage of predicted of the diffusion capacity of the lung for carbon monoxide; ERA: endothelin receptor antagonist; mPpa: mean pulmonary artery pressure; PCWP: pulmonary capillary wedge pressure; PDE-5: phosphodiesterase 5; PVR: pulmonary vascular resistance; IPAH: idiopathic pulmonary arterial hypertension; SScPAH: systemic sclerosis-associated pulmonary arterial hypertension; TLC%: percentage of predicted total long capacity.

**Table 2:** Characteristics of SScPAH patients.

	Antibody- profile	Cause of death	SSc disease duration (yr)^§^	Survival after PAH diagnosis (yr)	Medication at time of death
1 LcSSc*	Anticentromere	RV failure	4	0,5	prostacyclin
2 LcSSc	Anticentromere	RV failure	12	0,75	prostacyclin
3 LcSSc	Anticentromere	RV failure	1	0,08	prostacyclin
4 LcSSc	Anticentromere	Iatrogenic abdominal bleeding due to ascites punction	1	3	ERA, PDE-5 inhibitor
5 LcSSc	ANA	Post- LTX	13	0,42	Prostacyclin

Abbreviations: ANA: antinucleolar antibody; ERA: endothelin receptor antagonist; LcSSc: Limited cutaneous SSc; LTX: lung transplantation; PDE-5: phosphodiesterase 5; RV: right ventricle; SScPAH: Systemic sclerosis-associated pulmonary arterial hypertension. *According to [13]. ^§^Since first non-Raynaud symptom, at time of diagnosis of pulmonary arterial hypertension. Ascites caused by RV failure.
